# Investigation on the Gas-Phase Decomposition of Trichlorfon by GC-MS and Theoretical Calculation

**DOI:** 10.1371/journal.pone.0121389

**Published:** 2015-04-09

**Authors:** Kezhi Jiang, Ningwen Zhang, Hu Zhang, Jianmei Wang, Mingrong Qian

**Affiliations:** 1 Key Laboratory of Organosilicon Chemistry and Material Technology, Hangzhou Normal University, Hangzhou, Zhejiang, China; 2 MOA Key Lab for Pesticide Residue Detection, Institute of Quality and Standard for Agro-products, Zhejiang Academy of Agricultural Sciences, Hangzhou, Zhejiang, China; University of Calgary, CANADA

## Abstract

The gas phase pyrolysis of trichlorfon was investigated by the on-line gas chromatography – mass spectrometry (GC-MS) pyrolysis and theoretical calculations. Two reaction channels were proposed in the pyrolytic reaction, by analyzing the detected pyrolytic products in the total ion chromatography, including 2,2,2-trichloroacetaldehyde, dimethyl phosphite, and dichlorvos. Theoretical calculations showed that there is an intramolecular hydrogen bond between the hydroxyl group and the phosphate O atom in trichlorfon, through which the hydroxyl H atom can be easily transferred to phosphate O atom to trigger two pyrolytic channels. In path-a, migration of H atom results in direct decomposition of trichlorfon to give 2,2,2-trichloroacetaldehyde and dimethyl phosphite in one step. In path-b, migration of H atom in trichlorfon is combined with formation of the O-P bond to give an intermediate, followed by HCl elimination to afford dichlorvos. Path-a is kinetically more favorable than path-b, which is consistent with the GC-MS results.

## Introduction

**Fig 1:**
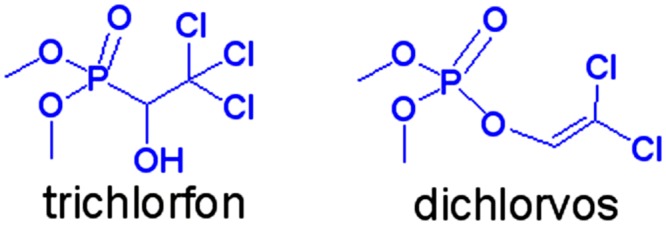
Structures of trichlorfon and dichlorvos.

Trichlorfon (**TCF**, Fig. 1) is an organophosphorus pesticide (OP) that is widely used to control agricultural pests because of its high insecticidal activity, acute toxicity, and relatively low environmental persistence [1–5]. It is difficult to remove OP from organic material using water, as it is for many other types of pesticide, and OP residues are often found on vegetable and fruit skins and even in groundwater. Methods have been developed to quantify **TCF** residues in fruits and vegetables [6] to allow food safety to be ensured.

Methods for analyzing OPs generally include gas chromatography (GC) [7,8] and high performance liquid chromatography (HPLC) [9,10]. Some OPs have been found to undergo pyrolysis in the heated GC injector port when they are analyzed by GC or GC-mass spectrometry (MS) [11–14]. **TCF** is a common OP but, unusually, it has no garlic-like odor. When **TCF** comes into contact with an alkaline medium it is converted into dichlorvos (**DCV**), which is actually much more toxic than **TCF** [15]. It is essential to investigate the thermostability and pyrolytic behavior of **TCF**, so that unnecessary errors can be avoided when **TCF** is analyzed in environmental media. Herein, we present a detailed mechanistic investigation of the decomposition pathways of **TCF** when it is analyzed by GC-MS.

## Materials and Methods

### Chemicals

Trichlorfon (**TCF**, *O*,*O-*dimethyl-2,2,2-trichloro-1-hydroxyethylphosphonate, 98.8%) and dichlorvos (**DCV**, *O*,*O*-dimethyl-*O*-2,2-dichlorovinylphosphate, 99.1%) were provided by the Shanghai Pesticide Research Institute (Shanghai, China). Methanol (HPLC grade) was obtained from Merck (Darmstadt, Germany).

### GC-MS experiments

The GC-MS experiments were performed using a Trace 2000 GC/DSQ MS instrument (Thermo-Fisher Scientific, Waltham, MA, USA) equipped with an HP-5MS capillary column (30 m long, 0.25 mm id, 0.25 μm film thickness; Agilent Technologies, Santa Clara, CA, USA) and the NIST (V2.0) mass spectra library. Xcalibur software (Version 1.4; Thermo-Fisher Scientific) was used to control the GC-MS instrument and to acquire and process the data.

Unless otherwise stated, the GC conditions were as described next. The injector and transfer line temperatures were both 250°C and the carrier gas (Helium, 99.999%) was used at a constant flow rate of 1.0 mL min^-1^. The GC oven temperature program was 50°C for 2 min, increased at 15°C min^-1^ to 260°C, which was held for 5 min. Each sample was dissolved in CH_3_OH and 0.5 μL of the solution was injected into the GC.

### Theoretical calculations

The theoretical calculations were performed using the Gaussian 09 program [16]. Due to the great accuracy, middle cost and high popularity of the DFT functional [17], the equilibrium geometries of the target species were optimized at the B3LYP/ 6–311+G(d,p) level. The M06-2X functional were also used to optimize these species by at the 6–311+G(d,p) level for comparison, since it provides the better optimization for energetic of the internal hydrogen bonds and reaction barriers [17]. Each optimized structure was identified as the true energy minimum if imaginary frequencies were absent. Transition states were identified by the presence of a single imaginary vibration frequency and the normal vibrational mode. The transition states were further confirmed using intrinsic reaction coordinate calculations. The optimized structures were visualized using GaussView (Version 3.09) software. Vibrational frequencies and zero point energies (ZPE) for all the key species were calculated at the same level of theory. Scaled frequencies were not considered since the errors on the calculated thermodynamical properties are almost negligible at this theoretical level [14,18]. Data for the geometries of all of the structures that were determined are available in the Supporting Information (S1–S9 Tables).

## Results and Discussion

### GC-MS analysis

**Fig 2:**
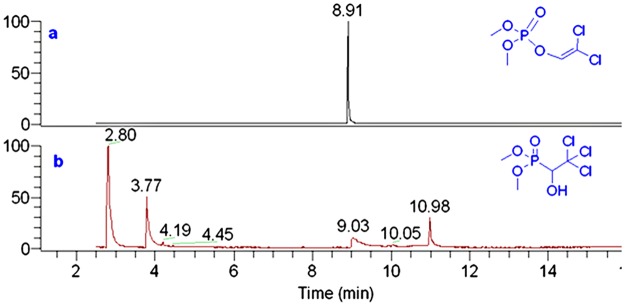
TIC of dichlorvos (a) and trichlorfon (b) by GC-MS.

Total ion current chromatograms (TIC), obtained using the GC-MS system, for **DCV** and **TCF** standards are shown in Fig. 2-a and Fig. 2-b, respectively. As can shown in Fig. 2-a, only a peak at *t*_*R*_ 8.91 min, corresponding to **DCV**, was observed in the TIC. Nevertheless, four major components were observed in Fig. 2-b. By comparing with the standard spectra in the NIST library, the components at *t*_*R*_ 2.80 min, *t*_*R*_ 3.77 min, *t*_*R*_ 9.03 min and *t*_*R*_ 10.98 min were identified as 2,2,2-trichloro- acetaldehyde (**TCA**), dimethyl phosphite (**DMP**), **DCV** and **TCF**, respectively.

**Fig 3:**
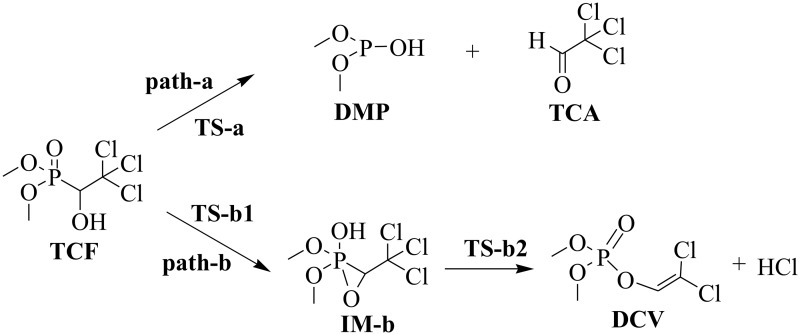
The proposed pyrolysis pathways of trichlorfon.

It is noteworthy that the sum of the molecular masses of **TCA** (C_2_HCl_3_O, 146 Da) and **DMP** (C_2_H_7_O_3_P, 110 Da) is identical to that of **TCF** (C_4_H_7_Cl_3_O_2_P, 256 Da). The mass difference between **DCV** (C_4_H_7_Cl_2_O_4_P, 220 Da) and **TCF** was found to be the same as that of HCl (36 Da). Thereby, two reaction pathways (Fig. 3) were postulated to interpret the pyrolysis of **TCF** in the heated GC injector. In reaction pathway path-**a**, the gas phase decomposition of **TCF** affords **TCA** and **DMP**, and the pyrolytic products of path-**b** are **DCV** and HCl. The HCl product of path-**b** was eluted from the capillary column before the solvent (methanol), so was not detected in the TIC (which was acquired from 2.5 min to 21 min after injection). Interestingly, a small peak at *t*_*R*_ 6.04 min was observed in the TIC of **TCF** (Fig. 2-b), which corresponds to octamethylcyclotetrasiloxane, originating from the degradation of the capillary column catalyzed by the pyrolytic product (HCl) of trichlorfon. Comparison of the peak area of these pyrolytic products indicated that the reaction path-**a** was more favored than path-**b** under the GC-MS conditions.

**Fig 4:**
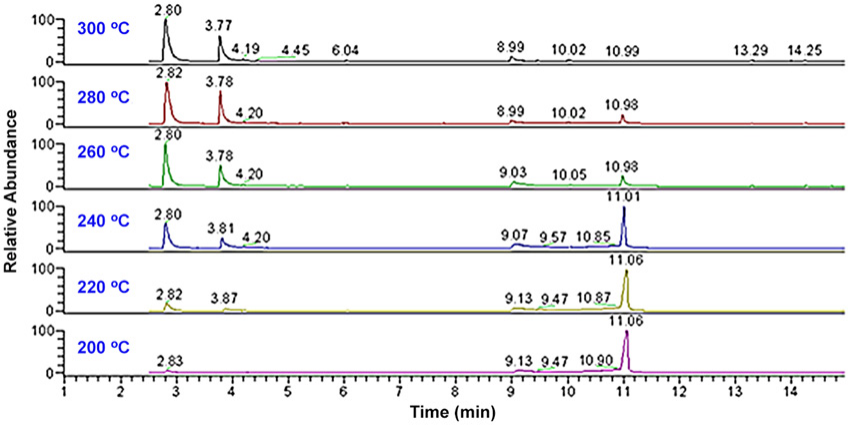
A series of TIC of trichlorfon with different GC injector temperatures.

To further investigate the effect of the temperature on the pyrolytic reaction, a series of online pyrolytic experiments were carried out for **TCF**, in which the GC injector temperature was set at 300°C, 280°C, 260°C, 240°C, 220°C, and 200°C, respectively. All the TIC were listed together in Fig. 4, in which the chromatographic peak at *t*_*R*_10.98 min (**TCF**) was almost not observed in the TIC acquired at the GC injector temperature of 300°C, indicating that almost all of the **TCF** underwent pyrolysis to mainly afford **TCA** and **DMP**. With reducing the injector temperature, an increasing peak area for **TCF** and a decreasing peak area for the products were obtained in Fig. 4.

### Fragmentation pathways

**Fig 5:**
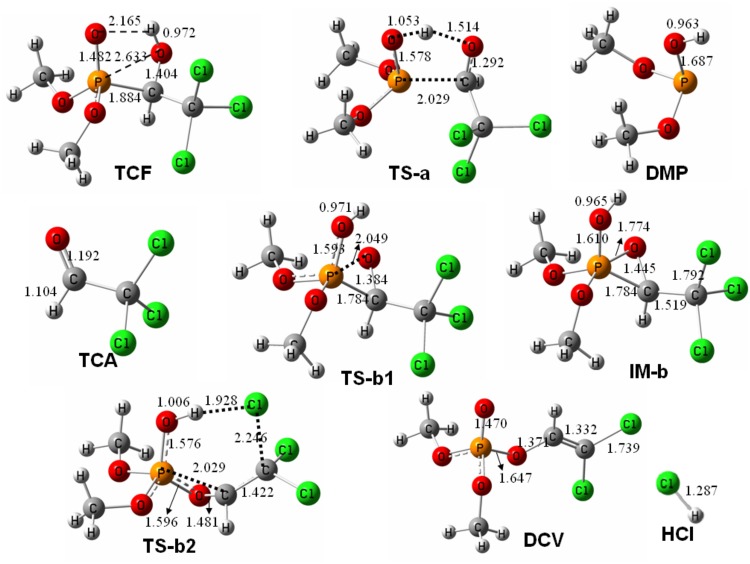
Optimized structures of the key species involved in the pyrolysis of trichlorfon at B3LYP/6-311+G(d,p).

The spectroscopic characteristics of **TCF** have been investigated previously [19], but the reactivity of **TCF** has not been investigated. We performed theoretical calculations to probe the mechanisms involved in the pyrolytic reactions of **TCF**. Full details of the structures of the species involved in the reactions were provided in Fig. 5, the corresponding thermodynamical parameters were summarized in Table 1, and data for the geometries of all of the structures are available in the Supporting Information. Noteworthy, one could notice that the energy barriers for both pathways are qualitatively similar at both B3LYP and M06-2X levels (Table 1), and thus only calculated results at 250°C (the pyrolytic temperature), obtained by at the B3LYP/6-311+G(d,p) level, were used for the following discussion.

**Table 1:** Enthalpies (H, in Hartree), relative Enthalpies (rel. H, in kJ mol^-1^), Free Energies (G, in Hartree) and relative Free Energies (rel. G, in kJ mol^-1^) of the structures, obtained at the B3LYP/6-311+G(d,p) level, the M06-2X/6-311+G(d,p) level, and the B3LYP/6-311+G(d,p) level (250°C), respectively.

**Structure**	B3LYP/6-311+G(d,p)				M06-2X/6-311+G(d,p)				B3LYP/6-311+G(d,p) level (250°C)			
	Enthalpies	rel. H	Free Energies	Rel. G	Enthalpies	Rel. H	Free Energies	Rel. G	Enthalpies	Rel. H	Free Energies	Rel. G
**TCF**	-2180.203324	0.0	-2180.265566	0.0	-2179.913378	0.0	-2179.972857	0.0	-2180.180463	0.0	-2180.319154	0.0
**TS-a**	-2180.174277	76.3	-2180.234233	82.3	-2179.877079	95.3	-2179.935367	98.4	-2180.152065	74.6	-2180.285892	87.3
**DMP**	-1532.686814		-1532.726466		-1532.543952		-1532.583338		-1532.677383		-1532.759169	
**TCA**	-647.498653		-647.541596		-647.332861		-647.374346		-647.485659		-647.577742	
**DMP + TCA**	-2180.185467	46.9	-2180.268062	-6.6	-2179.876813	96.0	-2179.957684	39.8	-2180.163042	45.7	-2180.336911	-46.6
**TS-b1**	-2180.159715	114.5	-2180.218204	124.3	-2179.868845	116.9	-2179.926339	122.1	-2180.13737	113.1	-2180.268795	132.2
**IM-b**	-2180.16076	111.8	-2180.219871	120.0	-2179.874036	103.3	-2179.932093	107.0	-2180.137759	112.1	-2180.271125	126.1
**TS-b2**	-2180.148549	143.8	-2180.207877	151.5	-2179.853262	157.8	-2179.911896	160.1	-2180.126191	142.5	-2180.259106	157.7
**DCV**	-1719.401163		-1719.459064		-1719.132785		-1719.190264		-1719.381131		-1719.508546	
**HCl**	-460.823873		-460.845066		-460.786232		-460.807418		-460.821375		-460.861827	
**DCV + HCl**	-2180.225036	-57.0	-2180.30413	-101.2	-2179.919017	-14.8	-2179.997682	-65.2	-2180.202506	-57.9	-2180.370373	-134.5

As is shown in Fig. 5, there is an intramolecular hydrogen bond (2.165 Å long), O···HO, between the hydroxyl group and the phosphate O atom in the optimized structure of **TCF**. The hydroxyl H atom can easily migrate to the phosphate O atom in the pyrolytic reaction pathways through the intramolecular hydrogen bond. In reaction pathway of path-**a**, migration of the hydroxyl H atom is accompanied by cleavage of the P-C bond (1.884 Å in **TCF** *versus* 2.029 Å in **TS-a**), leading to direct decomposition of **TCF** through a low energy barrier of 87.3 kJ/mol (**TS-a**).

In path-**b**, however, migration of the hydroxyl H atom is accompanied with the shrinkage of the distance between the P atom and the hydroxyl O atom to form a P-O bond (2.633 Å in **TCF** *versus* 2.049 Å in **TS-b1**). The process is completed through a low energy barrier of 132.2 kJ/mol (**TS-b1**), leading to an intermediate of **IM-b** with a three-membered ring [O, P, C], which has a hydroxyl group bonding to the P atom. **IM-b** has also been obtained in the TIC of **TCF** (the peak at *t*_*R*_ 10.05 min Fig. 2-b), which shares the same characteristic fragment ions (e.g. *m/z* 221, *m/z* 139, *m/z* 109) with **TCF** in EI-MS (S1 Fig.). The subsequent migration of a Cl atom to the hydroxyl H atom initiates HCl elimination from **IM-b** *via* the transition state **TS-b2**, and it results in the pyrolytic product of **DCV**. **DCV** is formed by opening the three-membered ring [O, P, C] *via* the breakage of the P-C bond (1.784 Å in **IM-b** *versus* 2.029 Å in **TS-b2**), and this has a relatively high energy barrier of 157.7 kJ/mol (**TS-b2**).

**Fig 6:**
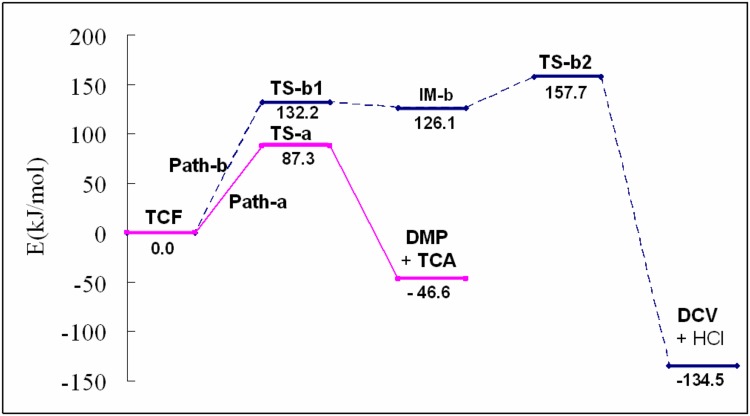
The schematic potential energy diagrams for the pyrolytic reactions of trichlorfon.

As shown in Table 1, the calculated results at the M06-2X/6-311+G(d,p) level revealed a relatively higher energy barrier than the corresponding one. However, both indicated similar results for the two competing reaction channels. Herein, the DFT calculated free energies (250°C) at the B3LYP/6-311+G(d,p) level were used for plotting the potential energy surface (Fig. 6) for the detailed discussion. The sum of HCl and **DCV** is located at 87.9 kJ/mol below that of **TCA** and **DMP** in the free energy, indicating that the products of path-b are thermodynamically more stable than those of path-a. Nevertheless, the energy barrier of path-**a** is much lower (by 70.4 kJ/mol) than that in path-**b**, indicating that path-**a** is much more kinetically favorable than path-**b** during the pyrolysis of **TCF**. Quantum calculations were performed herein to qualitatively describe the potential surface of the two reaction channels, and the results are in agreement with the experimental results. Further molecular dynamics simulations on the reactions [20–21] will be investigated in our future work.

## Conclusions

The gas phase pyrolysis of **TCF** was investigated in on-line GC-MS pyrolysis experiments and using theoretical calculations. The pyrolytic products 2,2,2-trichloro acetaldehyde, dimethyl phosphite, and **DCV** were detected in the TIC chromatogram of **TCF**. DFT calculations showed that there is an intramolecular hydrogen bond between the hydroxyl group and the phosphate O atom in **TCF**, and the hydroxyl H can easily be transferred to the phosphate O atom through this hydrogen bond. Migration of the H atom accompanied with cleavage of the P-C bond results in the formation of **TCA** and **DMP** in path-**a**. In path-**b**, migration of the H atom together with formation of the P-O bond results in an intermediate **IM-b**, which subsequently undergoes HCl elimination to afford **DCV**. Path-**a** is kinetically more favorable than path-**b**, and thus path-a occur more efficiently than path-b in the pyrolysis of **TCF** under the conditions in a GC injector. The results presented here provide us with a better understanding of the gas-phase pyrolytic reactions that occur during the GC-MS analysis of **TCF** and will allow analysts to avoid unnecessary errors during the GC or GC-MS analysis of **TCF**.

## Supporting Information

S1 Fig The EI-MS spectra of (a) dichlorvos and (b) the component at *t*_*R*_ 10.05 min. (TIF)

S1 Table Hard data on geometries for TCF obtained at the B3LYP/6-311+G(d,p) level. (DOC)

S2 Table Hard data on geometries for TS-a obtained at the B3LYP/6-311+G(d,p) level. (DOC)

S3 Table Hard data on geometries for DMP obtained at the B3LYP/6-311+G(d,p) level. (DOC)

S4 Table Hard data on geometries for TCA obtained at the B3LYP/6-311+G(d,p) level. (DOC)

S5 Table Hard data on geometries for TS-b1 obtained at the 3LYP/6-311+G(d,p) level. (DOC)

S6 Table Hard data on geometries for IM-b obtained at the B3LYP/6-311+G(d,p) level. (DOC)

S7 Table Hard data on geometries for TS-b2 obtained at the 3LYP/6-311+G(d,p) level. (DOC)

S8 Table Hard data on geometries for DCV obtained at the B3LYP/6-311+G(d,p) level. (DOC)

S9 Table Hard data on geometries for HCl obtained at the B3LYP/6-311+G(d,p) level. (DOC)
