# Supplementary figures and images for: Investigation on the Gas-Phase Decomposition of Trichlorfon by GC-MS and Theoretical Calculation
Source: PLoS One. 2015 Apr 9;10(4):e0121389. doi: 10.1371/journal.pone.0121389 (PMC4391870; doi:10.1371/journal.pone.0121389)

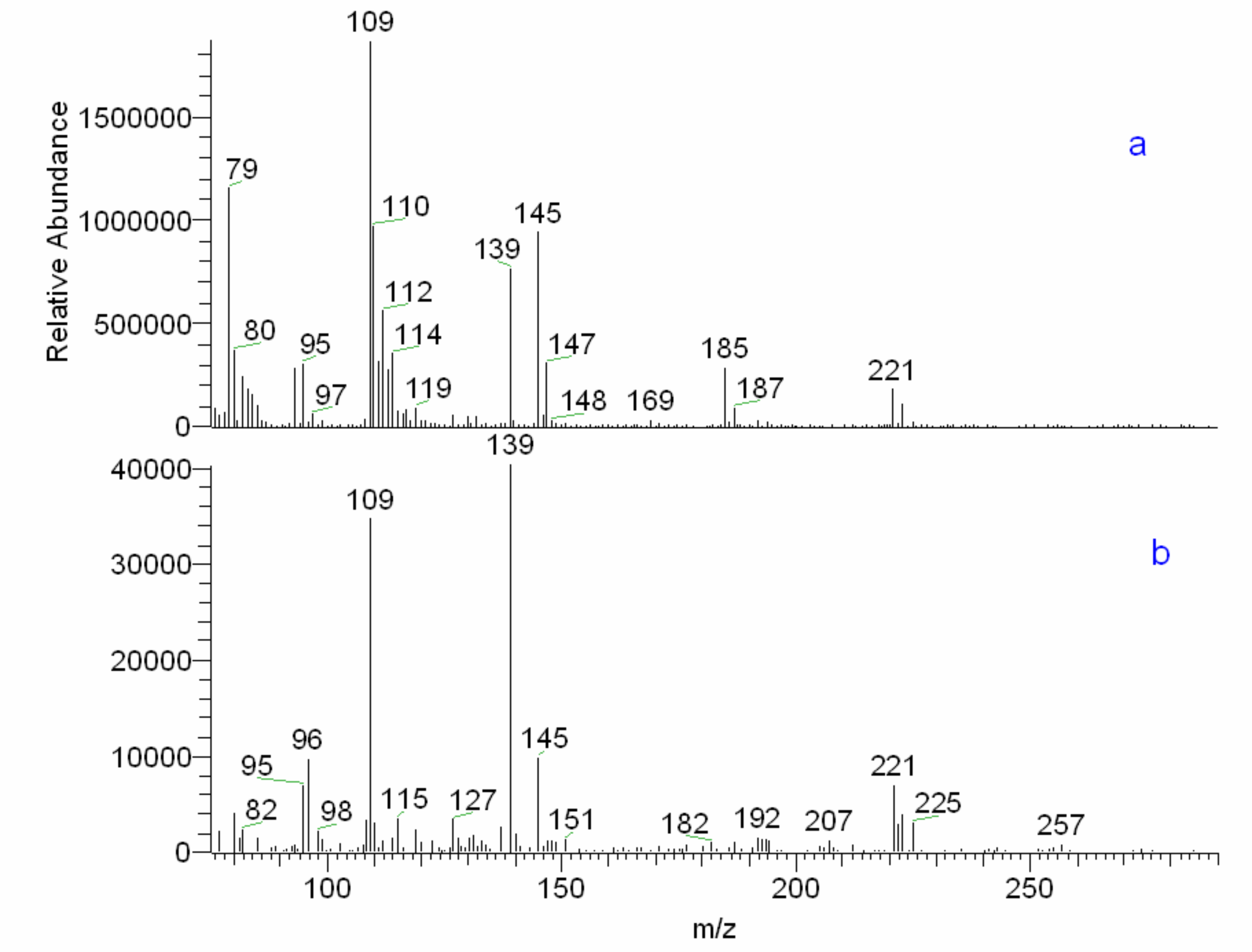

Supplement: S1 Fig — (TIF) [file pone.0121389.s001.tif]
