# Supplementary material for: Investigation on the Gas-Phase Decomposition of Trichlorfon by GC-MS and Theoretical Calculation
Source: PLoS One. 2015 Apr 9;10(4):e0121389. doi: 10.1371/journal.pone.0121389 (PMC4391870; doi:10.1371/journal.pone.0121389)
Supplement: S1 Table — (DOC) [file pone.0121389.s002.doc]

**S1 Table. Hard data on geometries for TCF obtained at the B3LYP/6-311+G(d,p) level.**

| Center Number | Atomic Number | Atomic  Type | Coordinates (Angstroms) | | |
| --- | --- | --- | --- | --- | --- |
|  |  |  | X | Y | Z |
| 1 | 6 | 0 | 3.284624 | -1.464635 | -0.991515 |
| 2 | 8 | 0 | 2.536099 | -0.243772 | -0.782192 |
| 3 | 15 | 0 | 1.426774 | -0.117579 | 0.383208 |
| 4 | 6 | 0 | -0.153667 | -0.754754 | -0.421260 |
| 5 | 6 | 0 | -1.477456 | -0.003486 | -0.111851 |
| 6 | 17 | 0 | -2.848255 | -0.935541 | -0.815005 |
| 7 | 8 | 0 | 1.672729 | -0.927094 | 1.600459 |
| 8 | 8 | 0 | 1.344343 | 1.453653 | 0.601187 |
| 9 | 6 | 0 | 1.956800 | 2.487444 | -0.198918 |
| 10 | 8 | 0 | -0.229419 | -2.112498 | -0.073620 |
| 11 | 17 | 0 | -1.440674 | 1.618851 | -0.906929 |
| 12 | 17 | 0 | -1.737523 | 0.182870 | 1.651334 |
| 13 | 1 | 0 | 4.139497 | -1.191380 | -1.606944 |
| 14 | 1 | 0 | 2.667587 | -2.197357 | -1.515307 |
| 15 | 1 | 0 | 3.618639 | -1.871118 | -0.036659 |
| 16 | 1 | 0 | -0.030805 | -0.694198 | -1.505212 |
| 17 | 1 | 0 | 3.029144 | 2.315381 | -0.282546 |
| 18 | 1 | 0 | 1.762249 | 3.417689 | 0.330425 |
| 19 | 1 | 0 | 1.502117 | 2.520503 | -1.189576 |
| 20 | 1 | 0 | 0.107828 | -2.210638 | 0.832476 |
