# Supplementary material for: Investigation on the Gas-Phase Decomposition of Trichlorfon by GC-MS and Theoretical Calculation
Source: PLoS One. 2015 Apr 9;10(4):e0121389. doi: 10.1371/journal.pone.0121389 (PMC4391870; doi:10.1371/journal.pone.0121389)
Supplement: S2 Table — (DOC) [file pone.0121389.s003.doc]

**S2 Table. Hard data on geometries for TS-a obtained at the B3LYP/6-311+G(d,p) level.**

| Center Number | Atomic Number | Atomic  Type | Coordinates (Angstroms) | | |
| --- | --- | --- | --- | --- | --- |
|  |  |  | X | Y | Z |
| 1 | 6 | 0 | -3.665842 | -1.335977 | 0.635297 |
| 2 | 8 | 0 | -2.219969 | -1.344419 | 0.530388 |
| 3 | 15 | 0 | -1.371878 | -0.194234 | -0.171476 |
| 4 | 6 | 0 | 0.462293 | -0.988946 | -0.519037 |
| 5 | 6 | 0 | 1.574370 | -0.103450 | 0.136062 |
| 6 | 17 | 0 | 3.152640 | -0.906145 | -0.138726 |
| 7 | 8 | 0 | -1.742783 | -0.018685 | -1.695364 |
| 8 | 8 | 0 | -1.898502 | 1.121860 | 0.582963 |
| 9 | 6 | 0 | -1.842925 | 2.468237 | 0.048901 |
| 10 | 8 | 0 | 0.456470 | -1.077380 | -1.808036 |
| 11 | 17 | 0 | 1.278652 | 0.027100 | 1.916495 |
| 12 | 17 | 0 | 1.608026 | 1.540618 | -0.593998 |
| 13 | 1 | 0 | -3.932654 | -2.281244 | 1.101862 |
| 14 | 1 | 0 | -4.111606 | -1.268580 | -0.358455 |
| 15 | 1 | 0 | -3.987189 | -0.501797 | 1.259111 |
| 16 | 1 | 0 | 0.417164 | -1.915845 | 0.081830 |
| 17 | 1 | 0 | -1.970331 | 2.453787 | -1.033299 |
| 18 | 1 | 0 | -0.891379 | 2.924727 | 0.317587 |
| 19 | 1 | 0 | -2.663846 | 3.005441 | 0.520405 |
| 20 | 1 | 0 | -0.879508 | -0.439906 | -2.127936 |
