# Supplementary material for: Investigation on the Gas-Phase Decomposition of Trichlorfon by GC-MS and Theoretical Calculation
Source: PLoS One. 2015 Apr 9;10(4):e0121389. doi: 10.1371/journal.pone.0121389 (PMC4391870; doi:10.1371/journal.pone.0121389)
Supplement: S3 Table — (DOC) [file pone.0121389.s004.doc]

**S3 Table. Hard data on geometries for DMP obtained at the B3LYP/6-311+G(d,p) level.**

| Center Number | Atomic Number | Atomic  Type | Coordinates (Angstroms) | | |
| --- | --- | --- | --- | --- | --- |
|  |  |  | X | Y | Z |
| 1 | 6 | 0 | 0.857206 | 0.000007 | -1.282965 |
| 2 | 6 | 0 | -0.048068 | -0.000001 | -0.023511 |
| 3 | 17 | 0 | -1.076187 | -1.474367 | -0.147887 |
| 4 | 8 | 0 | 2.048304 | 0.000003 | -1.251246 |
| 5 | 17 | 0 | -1.076158 | 1.474390 | -0.147853 |
| 6 | 17 | 0 | 0.887426 | -0.000027 | 1.475876 |
| 7 | 1 | 0 | 0.262347 | 0.000016 | -2.213497 |
