# Supplementary material for: Investigation on the Gas-Phase Decomposition of Trichlorfon by GC-MS and Theoretical Calculation
Source: PLoS One. 2015 Apr 9;10(4):e0121389. doi: 10.1371/journal.pone.0121389 (PMC4391870; doi:10.1371/journal.pone.0121389)
Supplement: S4 Table — (DOC) [file pone.0121389.s005.doc]

**S4 Table. Hard data on geometries for TCA obtained at the B3LYP/6-311+G(d,p) level.**

| Center Number | Atomic Number | Atomic  Type | Coordinates (Angstroms) | | |
| --- | --- | --- | --- | --- | --- |
|  |  |  | X | Y | Z |
| 1 | 6 | 0 | 1.813627 | 1.041786 | 0.294109 |
| 2 | 8 | 0 | 1.148682 | 0.264554 | -0.715270 |
| 3 | 15 | 0 | -0.048623 | -0.839583 | -0.537447 |
| 4 | 8 | 0 | 0.205360 | -1.149637 | 1.100783 |
| 5 | 8 | 0 | -1.432072 | 0.045905 | -0.477620 |
| 6 | 6 | 0 | -1.683313 | 1.166497 | 0.383233 |
| 7 | 1 | 0 | 2.734483 | 1.407458 | -0.159444 |
| 8 | 1 | 0 | 2.043718 | 0.428783 | 1.166189 |
| 9 | 1 | 0 | 1.198471 | 1.892951 | 0.597393 |
| 10 | 1 | 0 | -1.344145 | 0.961848 | 1.401585 |
| 11 | 1 | 0 | -2.760733 | 1.330248 | 0.384433 |
| 12 | 1 | 0 | -1.186192 | 2.059149 | -0.005456 |
| 13 | 1 | 0 | -0.113904 | -2.022959 | 1.349807 |
