# Supplementary material for: Investigation on the Gas-Phase Decomposition of Trichlorfon by GC-MS and Theoretical Calculation
Source: PLoS One. 2015 Apr 9;10(4):e0121389. doi: 10.1371/journal.pone.0121389 (PMC4391870; doi:10.1371/journal.pone.0121389)
Supplement: S5 Table — (DOC) [file pone.0121389.s006.doc]

**S5 Table. Hard data on geometries for TS-b1 obtained at the B3LYP/6-311+G(d,p) level.**

| Center Number | Atomic Number | Atomic  Type | Coordinates (Angstroms) | | |
| --- | --- | --- | --- | --- | --- |
|  |  |  | X | Y | Z |
| 1 | 6 | 0 | 3.024772 | -1.796901 | -0.946422 |
| 2 | 8 | 0 | 2.633207 | -0.444993 | -0.602653 |
| 3 | 15 | 0 | 1.388379 | -0.006982 | 0.292185 |
| 4 | 6 | 0 | -0.122507 | -0.489824 | -0.523792 |
| 5 | 6 | 0 | -1.536432 | -0.028558 | -0.114426 |
| 6 | 17 | 0 | -2.718937 | -1.137674 | -0.897715 |
| 7 | 8 | 0 | 1.471319 | -0.444919 | 1.821289 |
| 8 | 8 | 0 | 1.756247 | 1.542760 | 0.522459 |
| 9 | 6 | 0 | 2.030529 | 2.436868 | -0.570983 |
| 10 | 8 | 0 | 0.321174 | -1.719335 | -0.067449 |
| 11 | 17 | 0 | -1.793186 | 1.652567 | -0.734352 |
| 12 | 17 | 0 | -1.798270 | -0.052909 | 1.658127 |
| 13 | 1 | 0 | 2.169347 | -2.352161 | -1.324994 |
| 14 | 1 | 0 | 3.428669 | -2.291204 | -0.061869 |
| 15 | 1 | 0 | 3.802526 | -1.686787 | -1.699469 |
| 16 | 1 | 0 | -0.082070 | -0.344171 | -1.615687 |
| 17 | 1 | 0 | 2.951785 | 2.142003 | -1.075794 |
| 18 | 1 | 0 | 2.145595 | 3.424102 | -0.128243 |
| 19 | 1 | 0 | 1.198937 | 2.453269 | -1.279917 |
| 20 | 1 | 0 | 1.002453 | -1.291669 | 1.904740 |
