# Supplementary material for: Investigation on the Gas-Phase Decomposition of Trichlorfon by GC-MS and Theoretical Calculation
Source: PLoS One. 2015 Apr 9;10(4):e0121389. doi: 10.1371/journal.pone.0121389 (PMC4391870; doi:10.1371/journal.pone.0121389)
Supplement: S6 Table — (DOC) [file pone.0121389.s007.doc]

**S6 Table. Hard data on geometries for IM-b obtained at the B3LYP/6-311+G(d,p) level.**

| Center Number | Atomic Number | Atomic  Type | Coordinates (Angstroms) | | |
| --- | --- | --- | --- | --- | --- |
|  |  |  | X | Y | Z |
| 1 | 6 | 0 | 3.125805 | -1.666016 | -1.014332 |
| 2 | 8 | 0 | 2.689277 | -0.348827 | -0.623163 |
| 3 | 15 | 0 | 1.401204 | -0.035971 | 0.289252 |
| 4 | 6 | 0 | -0.130646 | -0.337548 | -0.574666 |
| 5 | 6 | 0 | -1.550890 | -0.054343 | -0.115653 |
| 6 | 17 | 0 | -2.632157 | -1.349226 | -0.768721 |
| 7 | 8 | 0 | 1.589807 | -0.358224 | 1.854974 |
| 8 | 8 | 0 | 1.709674 | 1.548275 | 0.511798 |
| 9 | 6 | 0 | 1.820645 | 2.441254 | -0.599896 |
| 10 | 8 | 0 | 0.500306 | -1.526590 | -0.048103 |
| 11 | 17 | 0 | -2.047452 | 1.536221 | -0.811823 |
| 12 | 17 | 0 | -1.726130 | -0.003918 | 1.667531 |
| 13 | 1 | 0 | 2.305415 | -2.234046 | -1.450808 |
| 14 | 1 | 0 | 3.524061 | -2.199151 | -0.148581 |
| 15 | 1 | 0 | 3.919665 | -1.506451 | -1.741757 |
| 16 | 1 | 0 | -0.117411 | -0.282253 | -1.666612 |
| 17 | 1 | 0 | 2.733584 | 2.237697 | -1.163951 |
| 18 | 1 | 0 | 1.859300 | 3.446550 | -0.183034 |
| 19 | 1 | 0 | 0.951400 | 2.365398 | -1.261336 |
| 20 | 1 | 0 | 1.201495 | -1.217615 | 2.061768 |
