# Supplementary material for: Investigation on the Gas-Phase Decomposition of Trichlorfon by GC-MS and Theoretical Calculation
Source: PLoS One. 2015 Apr 9;10(4):e0121389. doi: 10.1371/journal.pone.0121389 (PMC4391870; doi:10.1371/journal.pone.0121389)
Supplement: S7 Table — (DOC) [file pone.0121389.s008.doc]

**S7 Table. Hard data on geometries for TS-b2 obtained at the B3LYP/6-311+G(d,p) level.**

| Center Number | Atomic Number | Atomic  Type | Coordinates (Angstroms) | | |
| --- | --- | --- | --- | --- | --- |
|  |  |  | X | Y | Z |
| 1 | 6 | 0 | -2.937193 | 2.115382 | 0.052085 |
| 2 | 8 | 0 | -2.828082 | 0.702443 | -0.215749 |
| 3 | 15 | 0 | -1.428773 | -0.084836 | -0.076215 |
| 4 | 6 | 0 | 0.420529 | -0.468646 | -0.817543 |
| 5 | 6 | 0 | 1.691007 | -0.049514 | -0.335983 |
| 6 | 17 | 0 | 2.105958 | 1.643295 | -0.523510 |
| 7 | 8 | 0 | -1.074240 | 0.251111 | 1.422232 |
| 8 | 8 | 0 | -1.943817 | -1.563456 | -0.360000 |
| 9 | 6 | 0 | -3.297514 | -2.020635 | -0.094580 |
| 10 | 8 | 0 | -0.542693 | 0.580439 | -1.224585 |
| 11 | 17 | 0 | 3.001937 | -1.152816 | -0.738075 |
| 12 | 17 | 0 | 1.761713 | -0.231747 | 1.901111 |
| 13 | 1 | 0 | -2.286711 | 2.681786 | -0.617585 |
| 14 | 1 | 0 | -2.680491 | 2.321912 | 1.092176 |
| 15 | 1 | 0 | -3.976181 | 2.378217 | -0.136543 |
| 16 | 1 | 0 | 0.416605 | -1.319097 | -1.485539 |
| 17 | 1 | 0 | -3.523248 | -1.926735 | 0.968112 |
| 18 | 1 | 0 | -3.301128 | -3.068443 | -0.386507 |
| 19 | 1 | 0 | -4.010939 | -1.449790 | -0.685830 |
| 20 | 1 | 0 | -0.139954 | 0.032410 | 1.723939 |
