# Supplementary material for: Investigation on the Gas-Phase Decomposition of Trichlorfon by GC-MS and Theoretical Calculation
Source: PLoS One. 2015 Apr 9;10(4):e0121389. doi: 10.1371/journal.pone.0121389 (PMC4391870; doi:10.1371/journal.pone.0121389)
Supplement: S8 Table — (DOC) [file pone.0121389.s009.doc]

**S8 Table. Hard data on geometries for DCV obtained at the B3LYP/6-311+G(d,p) level.**

| Center Number | Atomic Number | Atomic  Type | Coordinates (Angstroms) | | |
| --- | --- | --- | --- | --- | --- |
|  |  |  | X | Y | Z |
| 1 | 6 | 0 | -2.092040 | 2.160870 | -1.008588 |
| 2 | 8 | 0 | -2.061203 | 0.763738 | -1.384080 |
| 3 | 15 | 0 | -1.684980 | -0.405560 | -0.382582 |
| 4 | 6 | 0 | 0.937418 | -0.595666 | -0.247948 |
| 5 | 6 | 0 | 2.134578 | -0.082728 | 0.029724 |
| 6 | 17 | 0 | 2.350227 | 1.368328 | 0.955298 |
| 7 | 8 | 0 | -2.606051 | -0.083158 | 0.878548 |
| 8 | 8 | 0 | -1.717927 | -1.754155 | -0.967047 |
| 9 | 8 | 0 | -0.206207 | 0.000279 | 0.217912 |
| 10 | 17 | 0 | 3.587791 | -0.861881 | -0.522570 |
| 11 | 1 | 0 | -2.874205 | 2.328598 | -0.267738 |
| 12 | 1 | 0 | -2.313786 | 2.706003 | -1.923180 |
| 13 | 1 | 0 | -1.121830 | 2.467392 | -0.614295 |
| 14 | 1 | 0 | 0.817734 | -1.503600 | -0.825652 |
| 15 | 6 | 0 | -2.589077 | -0.917674 | 2.059268 |
| 16 | 1 | 0 | -2.765603 | -1.959617 | 1.788243 |
| 17 | 1 | 0 | -1.632816 | -0.812804 | 2.574943 |
| 18 | 1 | 0 | -3.395257 | -0.554638 | 2.692641 |
