# Supplementary material for: Investigation on the Gas-Phase Decomposition of Trichlorfon by GC-MS and Theoretical Calculation
Source: PLoS One. 2015 Apr 9;10(4):e0121389. doi: 10.1371/journal.pone.0121389 (PMC4391870; doi:10.1371/journal.pone.0121389)
Supplement: S9 Table — (DOC) [file pone.0121389.s010.doc]

**S9 Table. Hard data on geometries for HCl obtained at the B3LYP/6-311+G(d,p) level.**

| Center Number | Atomic Number | Atomic  Type | Coordinates (Angstroms) | | |
| --- | --- | --- | --- | --- | --- |
|  |  |  | X | Y | Z |
| 1 | 17 | 0 | 0.000000 | 0.000000 | 0.071481 |
| 2 | 1 | 0 | 0.000000 | 0.000000 | -1.215183 |
